# The International Center for Alcohol Policies (ICAP) book series: a key resource globally for alcohol industry political strategies

**DOI:** 10.1186/s13011-023-00556-9

**Published:** 2023-08-18

**Authors:** Andrew Bartlett, Jim McCambridge

**Affiliations:** Department of Health Sciences, Seebohm Rowntree Building, University of York, Heslington, York, YO10 5DD UK

**Keywords:** Alcohol Policy, Alcohol Industry, Alcohol Science, Corporate social responsibility, Public Health Policy

## Abstract

This study examines the functions and purposes of the International Center for Alcohol Policies (ICAP) book series, published by Routledge between 1998 and 2010. The books were authored by invited academics, ICAP staffers, and alcohol industry representatives.

The key data source for this paper was the framing material – forewords, introductions, conclusions – of the books. A thematic analysis positioned the contents with regard to ongoing alcohol research and public health policy issues.

This was a project to ‘shift the paradigm’. ICAP frames alcohol policy choices in ways which direct policy attention to sub-groups rather than the population level. Population-level approaches are caricatured as ‘ideological’. The concept of ‘balance’ is prominent and is employed in multiple ways. Business interests are elided and industry involvement in policy making is promoted on scientific grounds. The intellectual programme is lent credibility by leading scientists and the imprimatur of an academic publisher.

While this attempt to change the paradigm in alcohol science has failed, ineffective alcohol policies remain common, uninformed by scientific evidence on how harms at the societal level may be reduced. The ICAP book series continues to serve its function as a resource to support the status quo in respect of alcohol policy.

## Introduction

The International Center for Alcohol Policies (ICAP), founded in 1995 by ten major alcohol companies, was part of the “steady increase in [alcohol] industry-funded “social aspects” and public relations organizations (SAPROs) that have been established to manage issues in areas that overlap with public health, such as alcohol control policies, medical research findings, and underage drinking” [1]. ICAP was unique in operating at the global level, as a counterpart of the World Health Organization (WHO) [2]. Other SAPROs operated in line with a plan forged by the leading alcohol companies to influence public relations and policy making at the country level [3]. Marcus Grant, a former scientist who had worked at the WHO from 1983 to 1994, was recruited to lead ICAP [2]. Grant was central to the organisation and orientation of the ICAP book series, editing four of the ten books, and contributing chapters, forewords, introductions etc. to several others.

The stated purpose of ICAP was: “To help reduce the abuse of alcohol worldwide and promote understanding of the role of alcohol in society. To encourage dialogue and pursue partnerships involving the beverage alcohol industry, the public health community and others interested in alcohol policy” [4]. ICAP claimed to strive for such public goods independently of industry-specific interests, adopting the tag line: “Analysis. Balance. Partnership.” Over the next 20 years, ICAP published an extensive number of briefing papers, issue reports, and reviews, as well as producing the book series examined in this paper. In 1997, ICAP were instrumental in drawing up the ‘Dublin Principles’ [5] which presented an outline for how academic researchers could work with industry and argued that policy makers and researchers should engage with research on the *benefits* of alcohol consumption in making policy. In 2014 ICAP merged with the Global Alcohol Producers Group (GAPG), a move described by Robaina and Babor as ICAP abandoning any pretence that it was not an industry lobby group, to form the International Alliance for Responsible Drinking (IARD) [6].

ICAP has been the subject of attention in the academic literature. Most importantly, Jernigan identified that it functioned, “like a WHO unit on alcohol” [2], and that “several ICAP publications seemed to counter or pre-empt similar WHO publications” [2]. However, Jernigan noted that unlike WHO work on alcohol, the emphasis was on patterns of drinking rather than population levels of consumption, as well as on the benefits of alcohol consumption. Jernigan wrote that ICAP, “provided model national and global alcohol policies based on the least effective strategies” [2], and that ICAP’s publications “were distinguished not by what was in them, …but by what was not: they excluded or attempted to refute evidence regarding the most effective strategies to reduce and prevent alcohol-related harm” [2]. Engaging with peer reviewed science to influence the content of the evidence-base as well as to generate favourable public relations benefits was a strategy that had been developed by public relations specialists from the early 1950s onwards working with both tobacco companies and the spirits industry in the U.S. [3]. ICAP thus developed its approach based on accumulated knowledge in the U.S. and elsewhere. This paper complements the work by Jernigan by making accessible to the alcohol research community a close reading of the ICAP authored sections of the book series, the orientation and function of the book series.

## Methods

This is a qualitative study of the ICAP books series conducted through a thematic analysis [7, 8] of the ‘framing’ sections, comprising the prefaces, forewords, acknowledgements, introductions and conclusions rather than the main contents of the books themselves. This material was chosen as these sections, written by the editors and/or ICAP staff, identified the most salient content of these mostly multi-authored volumes and placed these issues in the context of the wider book series *as far as those responsible for the commissioning and creation of the book series see it*.

As Jernigan wrote, the contributions by academic authors recruited into the series, “included useful contributions to various aspects of alcohol studies” [2], and the motivations and interests of the academic contributors are not the object of this paper. Rather, we argue that analysis of the ‘communicative act’ of framing [9] the series provides insight into the function and purpose of these books for ICAP, and therefore, by extension, into the interests of the leading global alcohol companies at play in participating in scientific publishing. On occasions we have drawn on other chapters where the authorial voice appears to speak as ICAP or directly to ICAP priorities, and also in order to explore how ICAP uses particular chapters that have been referenced the ‘framing’ sections.

The authors are a sociologist of science with a background in qualitative research and the study of ‘fringe science’ [10], and public health scientist conducting research on the alcohol industry. The combination of these disciplinary hinterlands lends a particular perspective on questions of the way in which scientific legitimacy is constructed. This collaboration is part of the Transformative Research on the Alcohol industry, Policy and Science (TRAPS) research programme, the aim of which is “to provide an empirical foundation for developing an orientation within alcohol sciences towards the alcohol industry as an object of study” [11].

Inductive coding of the framing sections [12] of the series was conducted by the first author. This process identified three initial themes; “challenging the consensus”, “health benefits of alcohol”, and “balance and moderation”. A deductive approach then identified two further themes: “origin stories” and “statements of intent/function”. The final analytic narrative was the product of collaboration between the co-authors. The paper concentrates on the themes of “challenging the consensus” and “balance and moderation”. Of particular interest were the points at which these themes intersect with material coded “statements of intent/function”.

The study thus examines narratives of construction and organisation of the book series, and interrogates the ways in which these books were positioned with regard to existing alcohol research and public health policy issues. We have a particular interest in analysing the *function* of these books for ICAP (and by extension the industry), including tracing their anxieties about the way in which these books would be received, in order to gain appreciation of the ways in which scientific artefacts were, at least in part, designed to be used as political resources. By ‘industry’, we refer exclusively to the transnational alcohol producers who were the sponsors of ICAP.

There are 10 books in the series published by Routledge between 1998 and 2010. Table 1 provides a full list of these books, including the number of direct industry contributors involved. Almost all of these are still available on the Routledge website, having been republished in paperback and/or converted to e-book formats.

**Table 1:** The ICAP book series

Year	Title	Editors/Authors	#Contributors	#Direct Industry Contributors	Total Citations
1998	Drinking Patterns and Their Consequences	Marcus Grant and Jorge Litvak (eds)	28	6	311
1998	Alcohol and Emerging Markets [14]	Marcus Grant (ed)	19	4	76
1999	Alcohol and Pleasure: A Health Perspective	Stanton Peele and Marcus Grant (eds)	41	4	368
2000	Drinking Occasions: Comparative Perspectives on Alcohol and Culture [16]	Dwight Heath	1	0	384
2001	Learning About Drinking [17]	Eleni Houghton and Ann Roche (eds)	18	3	163
2004	Moonshine Markets: Issues in Unrecorded Alcohol Beverage Consumption [18]	Alan Haworth and Ronald Simpson (eds)	24	3	207
2004	Reasonable Risk: Alcohol in Perspective [19]	Marjana Martinic and Barbara Leigh	2	1	9
2005	Corporate Social Responsibility and Alcohol: The Need and Potential for Partnership [20]	Marcus Grant and Joyce O’Connor (eds)	20	11	53
2008	Swimming With Crocodiles: The Culture of Extreme Drinking [21]	Marjana Martinic and Fiona Measham (eds)	26	5	230
2010	Expressions of Drunkenness (Four Hundred Rabbits) [22]	Anne Fox and Mike MacAvoy (eds)	12	3	22

Included in this are only authors who were directly employed by global alcohol producers, ICAP and industry trade associations. This count therefore excludes those working for social aspects organizations notwithstanding their origins [3]. The citation counts in this table are based on Google Scholar searches for both the book and individual chapters as of February 1st 2022.

## Results

### Constructing the book series

The origin of the book series can be traced to an ICAP meeting in September 1995 at which *Drinking Patterns and Their Consequences* was conceived. This book provides a list of its “editorial advisory group” and “external review panel” [13], an insight into the generative process not found in most other books in this series. These bodies were made up of academics and industry representatives - many of whom would go on to contribute to other books in the series as authors and/or editors. Perhaps most notable was the membership of former senior employees of the US National Institute of Alcohol Abuse and Alcoholism (NIAAA) Leland Towle and Loren Archer.

Several books were the product of conferences or meetings. The structure of *Alcohol and Pleasure* (1999), edited by Grant and Stanton Peele (a psychologist who argues against the medicalisation of addiction), mirrors that of the 1998 conference on which it was based (with, for example, ‘rapporteurs reports’) [15]. Academics associated with ARISE (Associates for Research into the Science of Enjoyment), a front group for the tobacco industry [23] were heavily involved in this conference, with David Warburton (University of Reading), the leading figure in ARISE, on the International Advisory Board for the conference and subsequent book, to which ARISE associated authors contributed four chapters [24–27].

Other books in the series owe their origins to ICAP organised conferences or meetings. *Moonshine Markets* (2004), edited by Alan Haworth (University of Zambia) and Ronald Simpson, the former VP of Corporate Scientific Affairs at Seagram, is described as the “outgrowth of a meeting the International Center for Alcohol Policies (ICAP) hosted in 1999, which brought together public health experts from developing countries to identify research topics of particular interest to these countries” [28]. *Corporate Social Responsibility and Alcohol* (2005), edited by Marcus Grant and Joyce O’Connor (National College of Ireland), is described as the product of a conference organised by ICAP and the National College of Ireland in 2002, though: “At least a third of the material included in this volume was commissioned after the conference, and many of the papers included were significantly rewritten to reflect our focus on the theme of corporate social responsibility” [29]. *Expressions of Drunkenness* (2010), the final volume in the series, edited by Anne Fox, an consultant with close ties to the alcohol industry [30], and Mike MacAvoy, the former chief executive of the SAPRO DrinkWise Australia, was the product of a workshop held in Paris in 2007.

The role of ICAP in shaping the ‘form’ of the books in the series is most obvious when they are the product of meetings and conferences they have arranged. ICAP’s roles in the design of other volumes is clear in other ways, including the role of ICAP employees such as, Marjana Martinic, Eleni Houghton, Jennifer Kast, and Daniya Tamenderova in editing or authoring material across the series. For example, *Learning About Drinking* (2001), edited by Houghton and Anne Roche (Flinders University), was the product of an ICAP-led initiative described as follows:

“The book is the result of a careful process of international consultation. In 1997, ICAP commissioned four papers by social scientists from Latin America, Australasia, South Africa, and Europe to capture their perceptions of how youth in those cultures learned about drinking alcohol. Each consultant was asked to focus particularly on the role of family, peers, culture, and religion. They were also asked to consider the effect more formal policies and programs have on learning about alcohol. These papers were discussed at a meeting held at ICAP in February 1998, which the four consultants attended, together with other education specialists and a representative from the alcohol beverage industry. Shortly after that meeting, a fifth paper on China was also commissioned. [...] Following the recommendations of the meeting, experts in relevant fields were invited to contribute chapters to this book. The five commissioned papers were made available to them all as source material and remained an important anchor point during the process of drafting, revising, and finalizing the chapters” [31].

This extract demonstrates the layers of influence that ICAP employed to shaping the contents of the book, laying bare both some of the work, and obfuscations, involved. The book chapters, contributions to the ‘constitutive forum’ [32] of alcohol science, are the result of a process in which the alcohol industry was closely and continuously involved.

Dwight Heath’s ‘Drinking Occasions’ (2000) - the only sole-authored book in the series and one of only two which is not an edited collection - was explicitly the result of an ICAP “consultancy”. Heath, a leading anthropologist on alcohol at Brown University, had contributed to all three earlier books in the series.

### Shifting paradigms in science and policy

The intentions behind the book series vis-à-vis the existing state of alcohol science are set out clearly in the closing paragraph of the concluding chapter (written by Grant and Eric Single, of the Canadian Centre on Substance Abuse/University of Toronto) of the first book in the series:

“In this book, the authors have attempted to provide a strong rationale for a paradigm shift from per capita consumption toward drinking patterns as a more effective basis for measuring and assessing alcohol-related problems and for more effective policy and prevention approaches […] The main purpose of this book is to contribute to the process of establishing and giving credibility to the concept of drinking patterns as a basis for alcohol policy” [33].

Similarly, Grant’s Preface to *Alcohol and Pleasure* states that “the principal factor [in deciding to organise the conference upon with the book was based] was the sense that the time had come to turn a new page in the long story of alcohol and society” [34], while in his Foreword to *Drinking Occasions*, the book series is presented as a coherent intellectual programme: “The first three volumes in this ICAP series on Alcohol in Society are multiauthored volumes dealing with shifting paradigms in alcohol studies” [35].

The early books are thus self-conscious interventions in the realm of science, ambitiously designed to change the terms of the debate – to move alcohol science and policy onto the ground preferred by ICAP. This took advantage of the under-development of the study of drinking patterns [36] and the rise of harm reduction ideas for drugs in the preceding decade [37, for a thoughtful discussion of the relationship between alcohol control and harm reduction, see 38], which had ready applications to alcohol [39]. Attention to drinking patterns, i.e. *how* people drink alcohol, is compatible with attention to *how much* they drink. Reducing overall alcohol consumption in order to reduce alcohol harm is not, however, in the interests of the industry [40]. The explicit purpose of the ICAP book series was to shift the paradigm in alcohol research from a focus on reducing consumption to making drinking patterns the central object of scientific and policy attention. In this, ICAP was directly engaging with unresolved scientific issues, predicated on clear understanding of the policy implications.

The narrative that ICAP research on ‘drinking patterns’ was shifting the paradigm in alcohol science is one that flows through the early books in the series. For example, the Introduction (subtitled “Drinking Patterns and Policy Development”) to *Alcohol and Emerging Markets* states that “There is now widespread support” [41] for the propositions set out in *Drinking Patterns and Their Consequences*, which was published the previous year. Similarly, the Introduction to *Moonshine Markets* by Haworth and Simpson [42] contains a section on “Patterns of Drinking”, referencing three specific chapters in *Drinking Patterns and Their Consequences*; those by Dwight Heath [43], by Amelia Arria (Johns Hopkins University) and Michael Gossop (National Addiction Centre, UK) [44], and one jointly authored by Wilson Acuda (University of Zimbabwe) and Barton Alexander (of Coors Brewing Company) [45].

A focus on the harms caused by alcohol in much of the public health literature provides a target for ICAP. Roche laments in *Learning About Drinking* that, “Comparatively little has been published about alcohol that has not been written from the perspective of its negative consequences” [46]. In *Alcohol and Pleasure*, Grant’s ‘Conclusion’ remarks that:

“Although the existence of beneficial effects is generally recognized, little attention has been paid to them within the public health context […] Even more modest than research on benefits is any existing attempt to describe beneficial patterns of drinking” [47].

The attempt to bring attention to the benefits of drinking involves a critique of the entire public health approach to alcohol, not just that of alcohol science.

A focus on drinking patterns and the benefits of drinking were two key themes for ICAP. Bringing the two together allows claims such as “much drinking is either entirely or virtually harm free” [33], a line in the first book repeated verbatim in the ‘Introduction’ to the second, *Alcohol and Emerging Markets*, [41], and a sentiment that is a significant focus of the third, *Alcohol and Pleasure*. A shift from population level approaches to a primary focus on ‘drinking patterns’ is a move that restricts attention to certain kinds of drinking and drinkers and admits consideration of harmless or even beneficial drinking. Many of the books include references to epidemiological claims that alcohol protects against heart disease, claims which rest on population level research, in which industry has been prominent [48]. However, the claims to the benefits of drinking extend well beyond the biological, the product of ICAP’s recruitment of anthropologists and sociologists. This is one example of the kind of “balance” that ICAP proposes; moving attention from harms to benefits. Grant writes in his ‘Foreword’ to Heath’s *Drinking Occasions*: “Having spent a quarter of a century working on alcohol policy issues, I am amazed at how little attention has been paid to the characteristics of the vast majority of drinking occasions, namely those which lead to feelings of subjective well-being and social cohesiveness. I see this book as an important contribution to this sorely neglected area” [35].

The power of this kind of narrative is that it can be seen as perfectly reasonable, drawing attention to a real limitation in the literature, even while being mobilised strategically with other arguments. For example, descriptions are coupled to prescriptions, such to promote ‘responsible’ drinking, that are not well rooted in research [49]. As the concluding line of *Learning About Drinking* has it: “If beverage alcohol is an enjoyable part of life for most adults, one should not be shy about encouraging responsible drinking and fully exploring mechanisms and pathways by which that may be achieved” [50]. That weaknesses in the science have led to poor alcohol policy is the key claim that runs through the ICAP books series.

### Alcohol public health science presented as a weary ideology

The per capita consumption paradigm is often dismissed as being less than scientific, despite the longstanding scientific consensus. Grant, Houghton, and Kast write in their ‘Introduction’ to *Alcohol and Emerging Markets* that, “the days are long gone when it was enough to mouth the words “less is better” as if they were some kind of religious mantra encapsulating divine truth.” [41]. Grant’s ‘Afterword’ to the same book contains the passage:

“In a sense, this is the book which I would have liked to have issued as a WHO report. It is intended to contrast with other publications which address alcohol problems in the developing world from particular ideological perspectives” [51].

The ICAP intellectual programme is presented as being particularly suitable for low and middle income countries, which have been a major focus of industry expansion, in contrast to high income countries with saturated markets [52, 53]. Grant writes here as if the existing paradigm was in the process of being swept away, with the alleged narrow “ideological” focus made redundant:

“…as I worked on […*Alcohol and Emerging Markets*] I got the sense of a new way of looking at drinking patterns and alcohol problems. There seemed to be a groundswell of scientific energy that acknowledged all the excellent research traditions of the past, but wanted to find a quite distinct approach to alcohol research that better fits the real needs and priorities of the developing world. What emerges is an impatience with the weary preoccupation with per capita consumption figures that often obscure more than they reveal” [51].

The framing of drinking patterns as a practical, pragmatic approach, explicitly or implicitly contrasted with the ‘ideological’ approach of the existing consensus, recurs throughout the series. In Grant and Single’s concluding chapter to *Drinking Patterns and Their Consequences* we have: “If less is always better (as the single-distribution approach assumes) then abstinence would logically be best of all. Harm-reduction measures, by contrast, presume that drinking will take place” [33]. Harm reduction is presented as “simply neutral regarding the long-term goal of interventions, which are pragmatic rather than ideological in their orientation. […] It is essentially a practical rather than idealized approach” [33]. Such themes draw on an intellectual hinterland of the development of earlier influential harm reduction ideas in respect of illegal drug use, which around this time were also being explored in relation to alcohol [39].

The authors close with: “A shift from a focus on overall consumption to patterns is therefore not merely of theoretical value. It represents a new pragmatism, which can lead to alcohol policies rooted in the interests of the individuals and communities they are intended to serve.” [33]. Society’s interests are presented as being advanced by this approach, whilst business interests play no explicit part in the narrative. In *Alcohol and Emerging Markets*, Grant, Houghton, and Kast write: “This book is intended to focus attention on the best available information from some other parts of the world, in order to highlight the importance of developing policies that are based on reality rather than ideology.” [41]. Similarly, Grant closes *Alcohol and Pleasure* by saying that the book:

“contribute[s] to a public policy that values education and information, that recognizes the autonomy of individuals and communities, and that rises above worn-out ideologies. The challenge is to build a scientifically sound approach that also will be of practical utility in making people’s lives healthier, of higher quality, and more pleasurable” [47].

Who could disagree with ICAP’s ‘pragmatic’, ‘practical’ and ‘scientifically-sound’ approach if we take their framing of the public health consensus at face value; as divorced from reality, ‘ideological’, ‘worn-out’, and making knowledge claims no more scientific than ‘religious mantra’?

### Offensive and defensive varieties of balance

Where books are not explicitly positioned as part of a programme to overthrow the paradigm, it is often suggested that their purpose is to provide *balance*. Sometimes, this is expressed in essentially defensive ways, a shield against anticipated criticism. Grant introduces *Drinking Occasions* by writing that while the book, “does not dwell upon drinking problems, […] It is therefore appropriate to acknowledge, here in the foreword to the book, that its intention is not to ignore or minimize the adverse health and social consequences of irresponsible drinking” [35]. Grant appears to be anticipating attacks on the legitimacy and purposes of the book:

“…it does not ignore alcohol related problems. What it does is put them in context. In any assessment of the place of alcohol in society, due weight must be given to the burden of disease and disability associated with it. But weight also needs to be given to its significant contribution to personal and social well-being. This book is intended to help balance the evidence” [35].

Calls for ‘balance’, in conjunction with the dismissal of population level approaches, sometimes takes a more ‘offensive’ form. In the ‘Rapporteur’s Report’ in *Alcohol and Pleasure*, Cynthia Chasokela (Zimbabwe Ministry of Health) and Eva Tongue (International Council on Alcohol and Addictions) write: “A conference consensus seemed to accept the role of a public health sector that develops and disseminates a neutral and scientifically well grounded body of health information” [54]. The implication is that existing information provided by the public health sector is neither neutral (what does ‘neutral’ mean with regard to alcohol harms) nor scientifically well-grounded. Chasokela and Tongue suggest an answer to why this may be: “Often, public health approaches are impeded by cultural baggage – such as the remnants of temperance traditions in some societies – that undermines the development of sound policies and the communication of scientific information about beverage alcohol” [54]. ‘Balance’ is required to counter the hidden moral agendas that shape public health research and policy making, agendas that need to be exposed in order to conduct better science and make better policy. As with other material used strategically by ICAP, such content can be seen to have some substance; for example, valuing health or welfare over other concerns does necessarily involve implicit judgements.

This appeal to ideas of ‘balance’ is not limited to intellectual approaches, but extends to ‘balancing’ the voices present in the debate. In their ‘Introduction’ to *Alcohol and Emerging Markets*, Grant, Houghton and Kast admit that:

“There are many reasons why those with a commitment to reducing alcohol-related problems might distrust the beverage alcohol industry […] No doubt there will be those who object to the inclusion of the industry perspective on ideological grounds, but we hope there will be many more who see it as expression of willingness to engage in a common mission where nobody, finally, has a monopoly on best practices” [41].

They continue, appealing to the reasonable reader “to judge for themselves the relative merits of an approach which includes the perspective of the beverage alcohol industry and an approach which excludes it. At the very least, it is to be hoped that scientists and scholars will always feel free to publish where they wish” [41]. This appeal is a defensive invocation of academic freedom, anticipating criticism of scientists’ engagement with the alcohol industry.

The claim is made that alcohol regulation is most effective when it is ‘balanced’, which is understood as “a balance between government regulation, industry self-regulation, and individual responsibility. And, in turn, what that requires is that all should be open to points-of-view other than their own” [41]. Evidence of *effective* regulation is largely absent from this narrative. The message discipline is impressive, as in for example Gaye Pedlow’s (Diageo, previously in PR at British American Tobacco) chapter in *Alcohol and Emerging Markets* which advances that, “alcohol policies need to be based upon an objective understanding of available research on alcohol use and misuse, and should aim to create a reasonable balance of government regulation, industry self-regulation, and individual responsibility” [55]. ICAP narrative is that the shortcomings of science and policy are in part the product of the exclusion of industry. Hurst Hannum (Tufts University) argues in his ‘Conclusion’ to *Corporate Social Responsibility and Alcohol* that this is an ethical imperative: “Being ethical and acting responsibly means being honest, transparent, consistent, and tolerant of those with different values. Only with such tolerance can there be mutual respect among those with different viewpoints” [56]. He goes on to say: “Adversarial relationships between the stakeholders only serve to weaken initiatives” [56]. Opposing industry involvement in policy making, as has been a key recommendation of WHO [57], is thus framed as morally problematic. Hannum, it should be noted, was the author of the ‘special commentary’ in Alcohol and Alcoholism that introduced the ICAP-supported ‘Dublin Principles’ to the wider alcohol research community [5].

As the series develops, the tone of these book’s position *vis à vis* the public health consensus becomes more moderate. By *Swimming With Crocodiles* (2008), which coincides with the development of the WHO global strategy on alcohol [58], the tone is more conciliatory. Discussing ‘stakeholder asymmetry’, Mark Leverton (Diageo) and Keith Evans (Drug and Alcohol Services, Australia, who was later revealed [59] to have worked as a consultant for ICAP in their efforts to shape alcohol policy in southern Africa) write: “Compounding this dichotomy is the perceived incompatibility between population-level measures related to alcohol and an emphasis on more targeted harm reduction. In fact, the two approaches are perfectly compatible” [60]. This is altogether less antagonistic than the material in the earlier books, and is reflected in more under-stated opposition to population-level measures by industry actors including ICAP’s successor organisation, the IARD [61]. Population-level approaches are no longer attacked as such, outright opposition to them is muted, and the emphasis is on involvement in policy making as the prize now to be won. Notwithstanding the failure of high-level attempts to shift the paradigms governing science and policy, involvement in policy making provides a key pathway to influence in national policy making, that has been pursued successfully [62].

## Discussion

In this paper we restrict our attention to the ICAP book series, and within that to the framing material of these books. The *scientific* content of the book series deserves its own attention as does the *impact* of the book series. This paper cannot address in detail questions of the book series’ contribution to the scientific literature and policy disputes/formation, nor, for example, the role of the book series in building a relationship between researchers and the alcohol industry (see papers by Mitchell and McCambridge [63, 64] for an exploration of the latter issue in a broader context). Nor has this paper considered the wider attempts by ICAP to contribute to the scientific literature, through its briefing papers and reports, and through funding individual researchers.

Nevertheless, this examination of the framing material of the ICAP books series reveals the purpose and *anxieties* of ICAP’s intellectual programme. Despite recruiting academics of some standing in alcohol research communities and lofty paradigm-changing rhetoric, we argue that the function of these books is as a resource to support alcohol industry interests, reinforced by the imprimatur of academic publication. As Jernigan and Mosher put it, “ICAP provides the industry with a scientific cover for its sales pitch” [65], and the ICAP book series was the apotheosis of these efforts. It involved credible researchers at prestigious institutions, publishing in the same spaces as, and sometimes with, industry representatives, in books shaped by ICAP staffers, endorsed by a respectable academic publisher. The contributions to the scientific literature made by the ICAP book series did not need to ‘overthrow the paradigm’ because ineffective alcohol policies uninformed by the scientific evidence from public health research on the per capita consumption paradigm remain the norm. Material such as the ICAP book series helps legitimate, but does not determine, this status quo. It has taken some time and considerable effort for this state of affairs to begin to change.

There is more to be written on the ICAP book series, beyond the scope of this paper, and more analysis to be done on ICAP’s broader programme. For example, the recruitment of anthropologists and other social researchers to develop an ICAP-supported literature on ‘drinking patterns’ is of some interest. Perhaps more importantly, the ICAP series involved the recruitment of authors based in the Global South, with books and chapters dedicated to addressing alcohol problems outside the Global North. This recruitment can be seen as part of ICAP’s focus on ‘emerging markets’, which led Jernigan and Mosher to describe the function this of aspect of ICAP’s work as providing, “the credentials the industry needs to convince governments of developing countries to neglect or weaken environmental safeguards regarding alcohol use” [65]. Bakke and Endal similarly identify the legitimating purpose of ICAP’s book, ‘Drinking in Context’, “where the emphasis rests upon the need to manage drinking patterns and strengthen industry/government/public health partnerships” [59].

## Conclusions

This paper is part of the TRAPS research programme [11], an interdisciplinary examination of the way in which the alcohol industry works to influence science and public policy. This focus was prompted by recognition by the WHO that alcohol industry influence was restricting the adoption what are understood by public health researchers to be the most effective measures to reduce alcohol harm [66].

This paper an analysis of only the framing of the ICAP intellectual project, a programme which carries implications for deeper understanding of corporate interventions in science. As suggested above, the recruitment by ICAP of academics from the Global South is particularly noteworthy, with researchers from those countries placed in an unenviable situation of worsening alcohol harms and limited access to scientific circuits of funding, dissemination, and reward. This is especially the case, given what we know of ICAP interventions in policy making in, for example, southern Africa [59].

While the ICAP book series did not succeed in changing the paradigm in alcohol science, it remains the case that alcohol policies often appear to be uninformed by scientific evidence on how harms at the societal level may be reduced [62]. The texts produced by academics and others for ICAP, and later, the IARD, serve as resources, providing scholarly and scientific justification for resisting public health research-informed policies. For example, while Working Together to Reduce Harmful Drinking [67], was not officially part of the ICAP book series (despite being published by Routledge), it was explicitly intended as a contribution to the WHO’s global strategy to reduce the harmful use of alcohol, and every chapter, barring the conclusion, made reference to material from the ICAP book series. The book series provides a corpus of citable ‘inscriptions’ [after 68] which “circulate in perpetuity in an economy of claim and credibility, disrupting knowledge claims” [69]. Corporate actors use publication in the scientific literature as a way to position themselves as credible participants in scientific and policy debates [see 70, 71].

Since the publication of the book series, ICAP has transformed into IARD, with overlap of staff and material, and with questions to be asked about the degree of continuity in not just their methods but also their strategy. IARD continues to make available ICAP resources on their website, as if they were their own. The enduring legacy of the ICAP book series, as attested to in the continuing availability of the publications, is as a resource in the broader political project. IARD have not been as prominently involved in making direct contributions to the scientific literature, and interestingly have evaded the scrutiny that was earlier directed towards ICAP. The space opened up for ‘partnership’ between industries, governments, and civil society presents a particular opportunity for activity [72]. Further examination of the ICAP-IARD transition, in particular the changes and continuities in presentation, and in scientific and political activities, as well as ongoing attention to IARD, will make valuable contributions to the contemporary alcohol policy evidence-base.

## Data Availability

The datasets used and/or analysed during the current study available from the corresponding author on reasonable request.
